# A Kriging based spatiotemporal approach for traffic volume data imputation

**DOI:** 10.1371/journal.pone.0195957

**Published:** 2018-04-17

**Authors:** Hongtai Yang, Jianjiang Yang, Lee D. Han, Xiaohan Liu, Li Pu, Shih-miao Chin, Ho-ling Hwang

**Affiliations:** 1 National United Engineering Laboratory of Integrated and Intelligent Transportation, School of Transportation and Logistics, Southwest Jiaotong University, Hi-Tech Industrial Development Zone, Chengdu, Sichuan, China; 2 Model Risk Management, Bank of America, Charlotte, NC, United States of America; 3 Department of Civil & Environmental Engineering, the University of Tennessee, Knoxville, TN, United States of America; 4 School of Architecture and Design, Southwest Jiaotong University, Hi-Tech Industrial Development Zone, Chengdu, Sichuan, China; 5 Center for Transportation Analysis, Oak Ridge National Laboratory, Cherahala Boulevard, Knoxville, TN, United States of America; Beihang University, CHINA

## Abstract

Along with the rapid development of Intelligent Transportation Systems, traffic data collection technologies have progressed fast. The emergence of innovative data collection technologies such as remote traffic microwave sensor, Bluetooth sensor, GPS-based floating car method, and automated license plate recognition, has significantly increased the variety and volume of traffic data. Despite the development of these technologies, the missing data issue is still a problem that poses great challenge for data based applications such as traffic forecasting, real-time incident detection, dynamic route guidance, and massive evacuation optimization. A thorough literature review suggests most current imputation models either focus on the temporal nature of the traffic data and fail to consider the spatial information of neighboring locations or assume the data follow a certain distribution. These two issues reduce the imputation accuracy and limit the use of the corresponding imputation methods respectively. As a result, this paper presents a Kriging based data imputation approach that is able to fully utilize the spatiotemporal correlation in the traffic data and that does not assume the data follow any distribution. A set of scenarios with different missing rates are used to evaluate the performance of the proposed method. The performance of the proposed method was compared with that of two other widely used methods, historical average and K-nearest neighborhood. Comparison results indicate that the proposed method has the highest imputation accuracy and is more flexible compared to other methods.

## Introduction

Along with the rapid development of Intelligent Transportation Systems (ITS), traffic data collection technologies have been evolving dramatically. [1, 2]. On the one hand, the emergence of innovative data collection technologies such as remote traffic microwave sensor (RTMS), Bluetooth sensor, and GPS-based floating car method have made traffic data collection much easier than before. [3–5].

Despite the development of technologies, the missing data problem still exists. Missing data could be due to various reasons such as malfunction of sensors and loss of communication. The Mobility Monitoring Program of the Texas Transportation Institute (TTI) reported that after screening erroneous data, the complete rate of collected data can be anywhere between 16% and 93% with a median value of 67% [6]. Williams and Hoel [7] reported that the data missing rate collected by Georgia’s statewide advanced traffic management system was 10% or higher. The data missing rate of the freeway Performance Measurement System (PeMS) in Los Angeles was found to be 15% [8]. Chandra and Al-Deek [9] reported a 15% missing rate of data collected by loop detectors on I-4 in Orlando, Florida. An empirical study showed that the average missing rate of data collected by Georgia NaviGAtor system at GA 400 was between 4% and 14% [10]. In Beijing, China, the average missing rate of the daily traffic volume data was about 10% (4% due to malfunction of detectors and 6% due to other reasons) with the missing rate of data collected by some detectors as high as 25% [11].

The missing data issue has posed great challenges for data based applications such as traffic forecasting, incident detection, route guidance, and massive evacuation optimization. Therefore, a lot of efforts need to be made to impute the missing data.

Most current imputation techniques could estimate a single value for the missing data point. These techniques include heuristic imputation, prediction imputation, and statistical learning imputation etc. The heuristic imputation methods fill the missing data point by averaging data of the same time period on neighboring days or averaging data of neighboring time periods of the same day. These methods are based on the assumption that traffic characteristics are similar at the same time period of different days or the fluctuations of traffic data are low during short time period [12]. Another group of heuristic methods are called pattern-similar imputation methods which search for the most similar traffic data series from historical data and use it to estimate missing data points [13]. These heuristic methods make good use of the similarity and periodicity of traffic data. However, the local variation and unexpected changes of traffic pattern could result in high imputation inaccuracy [14, 15]. To address this issue, two advanced methods, Bayesian Principal Component Analysis (BPCA) and Probabilistic Principal Component Analysis (PPCA), were recently proposed by Qu et al. [11, 16]. Researchers first show that traffic flow data follow Gaussian distribution and that principal component analysis (PCA) can be used to retrieve the features of traffic flow. Then, a robust PCA is used to filter out the abnormal traffic flow data that disturb the imputation process. The difference between BPCA and PPCA is that BPCA is slower than PPCA but yields similar results. BPCA is usually carried out first on a small sample to determine the important parameters. Then, the imputation tasks are performed by PPCA with those parameters.

Prediction method is also an important way to impute data. Regression method is a classic example. Al-Deek et al. [9] compared the feasibility and imputation accuracy of three regression models, multiple regression, time series, and pairwise regression. They found that quadratic model performed better because of its ability to capture nonlinear relationships among variables. To impute missing traffic data during holidays, Liu et al. introduced a new procedure using non-parametric regression, the K-nearest neighborhood (KNN) method, estimate missing values for different types of highways on holidays [17]. Other regression models that have been used for imputation include ARIMA [18], support vector regression [19], exponential smoothing [18], neural network [20], hidden Markov Model [21] and so on [12]. However, these prediction methods can only use data before the missing data point and ignore the data after the missing data, which means they cannot take full advantage of the data set for imputation.

Statistical learning imputation methods assume that data are missing at random. Specifically, the missing data are considered as realizations of random variables characterized by a certain probability distribution function. Antonio et al. proposed an incremental approach theoretically motivated by the Statistical Learning Theory of Vapnik, and provided a new paradigm for missing data imputation [22]. Ma et al. employed copula theory to build a connection between the correlation function and the marginal distribution function of traffic flow, and proved effectiveness of the method to impute missing data in large-scale transportation networks [23].

Most of the methods mentioned above only use temporal information for imputation, while spatial information is not well used. As traffic flows from upstream to downstream, the traffic stream characteristics at a certain location are usually closely related to those at neighboring locations. The incorporation of surrounding traffic information has been proved to be useful to improve traffic prediction accuracy [24–26]. Literature review results show that Markov chain Monte Carlo (MCMC) [10] and PPCA [11] are two representative methods that use both temporal and spatial information. However, both MCMC and PPCA methods assume a probability distribution model of the data [12]. This assumption limits the use of these methods since when the data does not follow a specific distribution these methods may generate inaccurate imputation results. As a result, this paper proposed an alternative method, a Kriging based method, that does not assume the data follow any probability distribution and that can fully use both temporal and spatial information, to impute data.

The rest of the paper is organized as follows. Section 2 describes the proposed Kriging based imputation approach and other benchmark models that are used for comparison. Study location and data are described in Section 3. A brief description of data missing patterns and missing ratios are presented in Section 4. Section 5 compares imputation accuracy of proposed approach with benchmark models, historical average and KNN. Concluding remarks are given in section 6.

## Methodology

### Kriging based spatiotemporal imputation approach

#### Background about Kriging

Kriging originated in the mining industry in the early 1950’s as a means of improving ore reserve estimation and has been used as synonym for geo-statistical interpolation for many decades. Traditionally, the Kriging method only deals with spatial variables. Consider a set of spatial data *z (μ*_*i*_*)* of an attribute *z* at location *U*_*i*_, *i = 1*, *2*, *3*, ..,*n*, where *U* is a vector of spatial coordinates *μ*_*i*_ *= (x*_*i*_, *y*_*i*_*)*. The task of data imputation is to estimate missing values of *z* at a set of *m* locations. Generally speaking, Kriging is just optimal interpolation method based on regression using observed surrounding data points, weighted according to covariance values. Compared with other methods, the Kriging method has following advantages: 1) It can reduce the effect of data clustering by assigning data points within a cluster less weight; 2) It can produce a measure for possible estimation error (Kriging variance), along with the estimation of the missing values [27].

#### Kriging based spatiotemporal imputation

Traffic stream characteristics change over time and space. Traffic volume at a location is not only correlated with the traffic volumes at upstream and downstream locations but also correlated with volumes of the previous and next time step [28]. Thus, time dimension needs to be considered in the Kriging model to better estimate the missing data.

In the example of traffic volume, each data point is referenced by its temporal timestamp *t*_*i*_, and spatial location *μ*_*α*_ *= (x*_*α*_, *y*_*α*_*)*. Different from the aforementioned traditional spatial Kriging models, the coordinates are simplified as *μ*_*α*_ *= x*_*α*_ as roads can be seen as a longitudinal system with only one spatial dimension, in which *x*_*α*_ is the mile marker.

In the space-time framework, traffic volume is formulated as *Q (μ*_*α*_, *t*_*i*_*); α = 1*, *2*, *…*, *n; i = 1*, *2*, *…*, *m*. Similar to the spatial models, the covariance is defined as the variance of the mean squared difference between data separated by a given spatial and temporal lag *(h*_*s*_, *t*_*s*_*)*:

$$C(h_{s},h_{t})=E[{\left(z(u_{\alpha },t_{\alpha })-z(u_{\alpha }+h_{s},t_{\alpha }+h_{t})\right)}^{2}]$$ (1)

To be consistent with the common practice in spatial statistics, experiment semivariogram is computed as half of covariance:

$${\hat{\gamma }}_{s,t}(h_{s},h_{t})=\frac{1}{2}E[{\left(z(u_{\alpha },t_{\alpha })-z(u_{\alpha }+h_{s},t_{\alpha }+h_{t})\right)}^{2}]$$ (2)

In the ordinary space-time Kriging system, the missing value Q^*^(μ, *t)* can be estimated as weighted average of values of surrounding locations:

$$Q^{*}(u,t)=\sum \lambda_{\alpha ,i}(u,t)Q(u_{\alpha },t_{i})\;with\;\sum \lambda_{\alpha }(u,t)=1$$ (3)

The weights *λ*_*α*, *i*_ *(μ*_*α*_, *t*_*i*_*)* assigned to each neighboring data point are calculated by minimizing the prediction variance:

$$\sigma^{2}(u,t)=Var[Q^{*}(u,t)-Q(u,t)],$$ (4)

while maintaining unbiasedness of the estimated value Q^*^(μ, *t)*.

As the calculation of covariance is based on both spatial and temporal distance between data points, the spatial and temporal correlations of traffic volumes are well considered and utilized in the model. In this study, the Gaussian variogram method is used to approximate empirical variogram in the proposed spatiotemporal imputation method. It should be noted that the temporal and spatial properties of data are not similar, which makes it difficult for the variogram to capture the temporal and spatial variability. To address this issue, the very straightforward solution is to regard time dimension as the third orthogonal dimension and to extend traditional 2-dimensional Kriging to a 3-dimensional Kriging. In addition, the temporal dimension has to be rescaled to align with the spatial directions. All the works mentioned above are implemented using R studio and related packages.

### Benchmark imputation methods for comparison

To evaluate the performance of the Kriging based spatiotemporal approach, the results were compared with those of two classical imputation models, historical average and KNN.

#### Historical average (same time and weekdays and same stations)

The historical average model is a widely used prediction model [29]. A missing data point is estimated by averaging data points of the same location at the same time of the day on the same day of the week. To be more robust to extreme values, the historical median can be used too.

#### K-nearest neighborhood

Because the data were recorded every 30 seconds by sensors, 2880 data points were collected every day (*2880* = 24h * 60min/h * 60s/min / 30s). In order to implement KNN method [30], the traffic volume data needs to be reformatted as a 2880×(s_*_ *d)* matrix), where *s* is the number of stations and *d* is total number of days. After the transformation, the data collected at a given station on a specific day is considered as a column of the matrix.

For the column with missing values, the Euclidean distances between this column and other columns were calculated to find *k* nearest neighbors. Finally, the weights for the *k* nearest neighbors were derived and the estimation of missing values were the weighted averages of k nearest neighbors [31].

### Evaluation criteria

Mean absolute deviation (MAD) and root mean squared error (RMSE) were used to compare imputation results of proposed approach with benchmark methods. Suppose there were *n* missing data points in the test dataset with $V_{act}^{i}$ as ground truth for *i*^*th*^ missing data point and $V_{est}^{i}$ as the estimated value for the missing data point. The two measures could be calculated as follows:

$$MAD=\frac{\sum_{i}^{n}\left|V_{act}^{i}-V_{est}^{i}\right|}{n}$$ (5)

$$RMSE=\sqrt{\frac{\sum_{i}^{n}{\left(V_{act}^{i}-V_{est}^{i}\right)}^{2}}{n}}$$ (6)

## Data source and study locations

Smart Way [32], a key program of Tennessee’s intelligent transportation system, uses solar-powered nonintrusive RTMS to collect real-time highway traffic information (including volume, speed and occupancy). The collected data are sent to the traffic management center. The data used in this study are collected by these RTMS radars installed along interstates across Tennessee. Vehicle presence, traffic volume, speed, and occupancy per lane are recorded every 30 seconds by these sensors [33].

**Fig 1:**
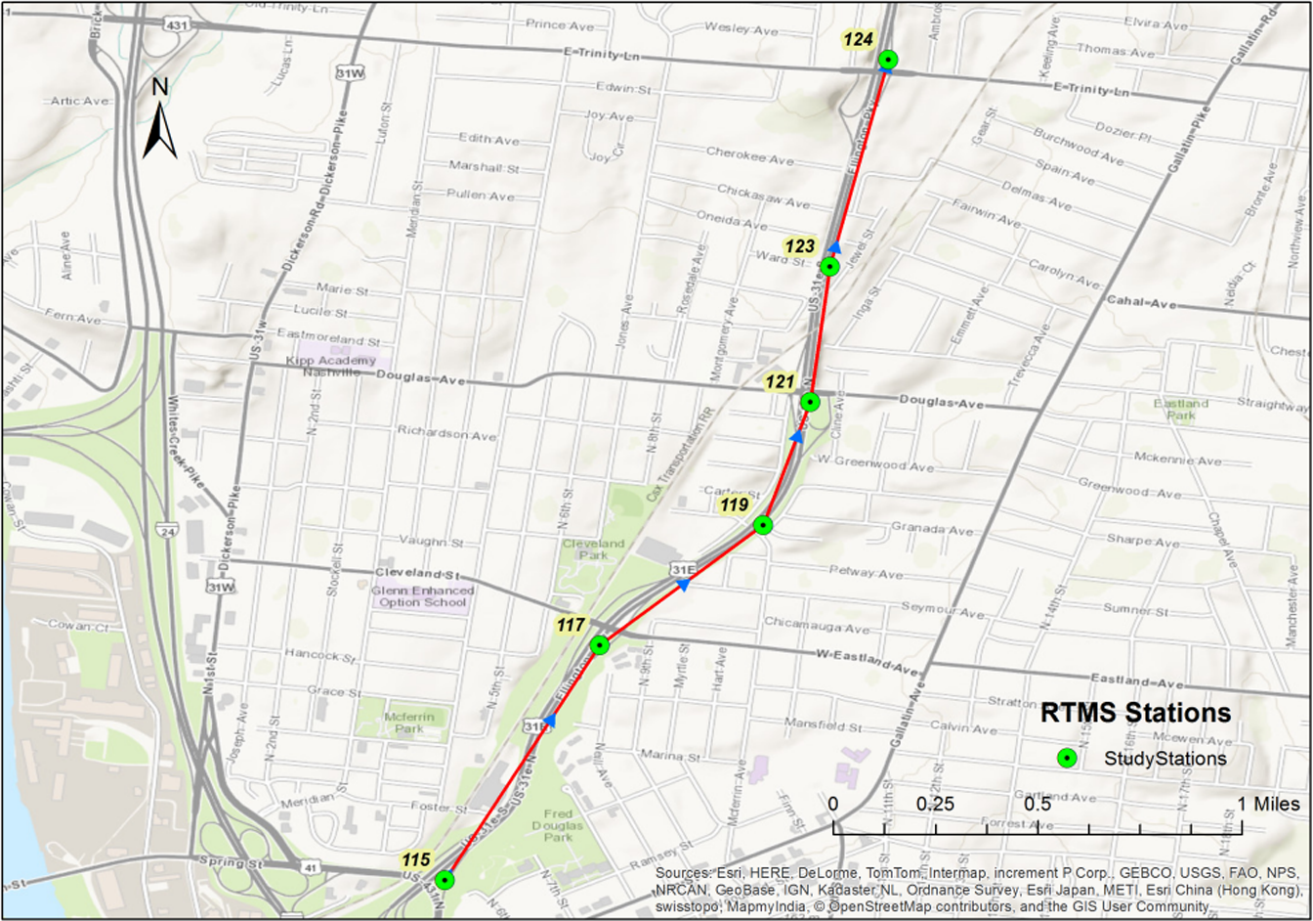
RTMS stations for this study.

To better identify data missing patterns, a long period of 33 days of data (from April 29 to May 31, 2013) are collected for six RTMS stations along Ellington Parkway in Nashville, Tennessee [34]. The detailed description of RTMS stations is presented in Table 1 and their locations are given in Fig 1. As the data are collected every 30 seconds, a total of 570,240 (2880×33×6) data points would be obtained if no data were missing.

**Table 1:** Description of RTMS stations.

Station	Direction	Location	Lanes	Mile marker
115	Northbound	Ellington Parkway @I24	2	10.6
117	Northbound	Ellington Parkway @Cleveland	2	11.4
119	Northbound	Ellington Parkway @Granada	2	11.8
121	Northbound	Ellington Parkway @Douglas Ave	2	12.2
123	Northbound	Ellington Parkway @South of Trinity	2	12.4
124	Northbound	Ellington Parkway @Trinity	2	13.0

Different from previous studies, imputation was performed on the raw data in this study instead of aggregated data to prevent information loss during the aggregation process. The data description and missing rates are shown in Table 2. Numbers in parenthesis indicate corresponding standard errors. The average count means the average number of vehicles that were recorded by sensors over 30 seconds.

**Table 2:** Data description.

Station number	Lane	Average speed (mi/h)	Average count	Missing rate
115	1	47.41 (22.67)	5 (4)	3.19%
	2	36.63 (31.47)	2 (3)	
117	1	38.46 (33.65)	3 (4)	33.83%
	2	25.94 (31.75)	2 (4)	
119	1	57.85 (21.71)	5 (4)	14.27%
	2	37.77 (27.38)	3 (5)	
121	1	41.95 (17.56)	5 (4)	15.28%
	2	40.47 (24.18)	4 (4)	
123	1	43.41 (26.52)	4 (5)	18.77%
	2	43.22 (26.63)	4 (5)	
124	1	40.06 (18.46)	4 (3)	17.28%
	2	35.26 (21.38)	3 (4)	

## Data missing rates

**Fig 2:**
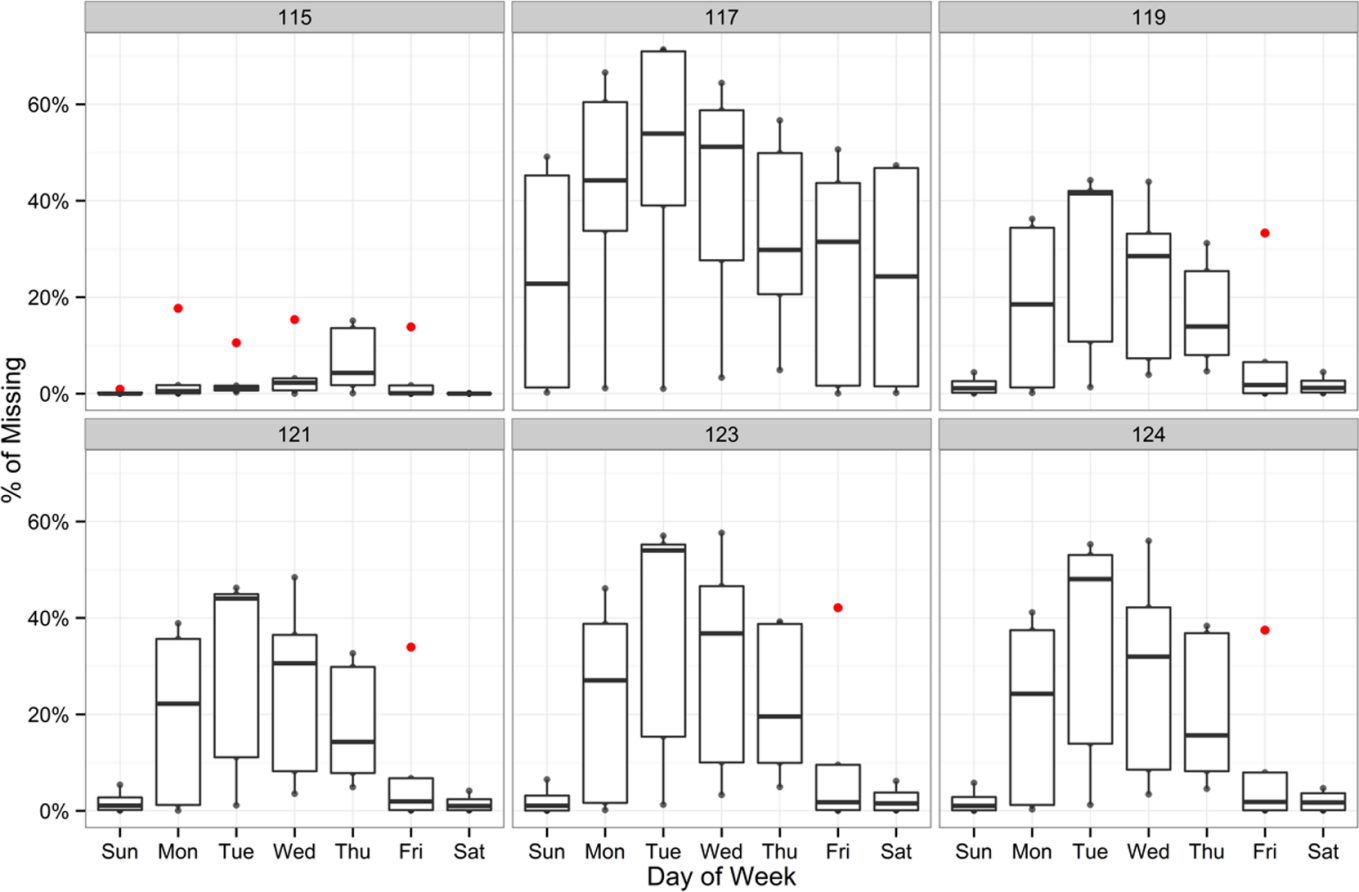
Boxplot of data missing rates by station and day of the week.

To understand RTMS radars’ performance, a boxplot of missing rates by station and day of the week is shown in Fig 2. It shows that the performance of a station varies across days and the performance of different stations on the same day also varies significantly. Station 115 usually has the lowest data missing rates with only a few exceptions. In contrast, station 117 has the highest data missing rates across the week.

## Evaluation of imputation performance

### Experiment design of data missing scenarios

A complete data set is preferred to train the proposed and benchmark models and to evaluate their performance. A close look at the data reveals that the data collected by station 119 on May 17, 2013 has a low data missing rate, 0.03% (only one data point is missing), and thus is used in this study. Another reason for choosing station 119 is that there are both upstream stations and downstream stations, which means there are both upstream and downstream information available.

To compare the imputation performance, imputation methods are tested based on simulated scenarios with different data missing rates. The missing rates are set to be different percentiles of the actual missing rates for all stations during the 33 days of the study. Also, the missing data points are generated randomly. The simulation process for the simulation is as follows:

1. Choose a specific data missing rate among 25%, 30%, 35%, …, 75% percentiles of missing rates of all stations during the study period;
2. Based on the missing rate selected above, generate the number of points to be flagged as missing in the dataset;
3. Generate missing data points randomly;
4. Repeat step 1 to 3 for different missing rates to generate corresponding scenarios;
5. Perform imputation on these generated scenarios using the proposed method and benchmark methods, and compare their results.

For the whole day of May 17, 2013, the traffic was congested during the rush hours and was in free-flow condition during the non-rush hours, just like the other days. Since the missing data points were generated randomly, with missing rate ranging from 1.0% and 36.1%, the missing data was likely to cover both free-flow conditions and congested conditions.

### Imputation performance

**Fig 3:**
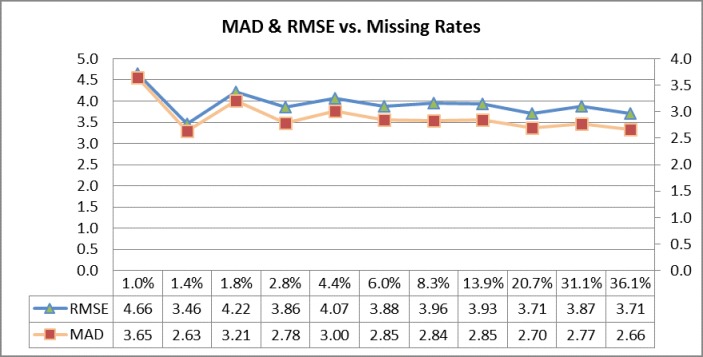
Initial variogram.

**Fig 4:**
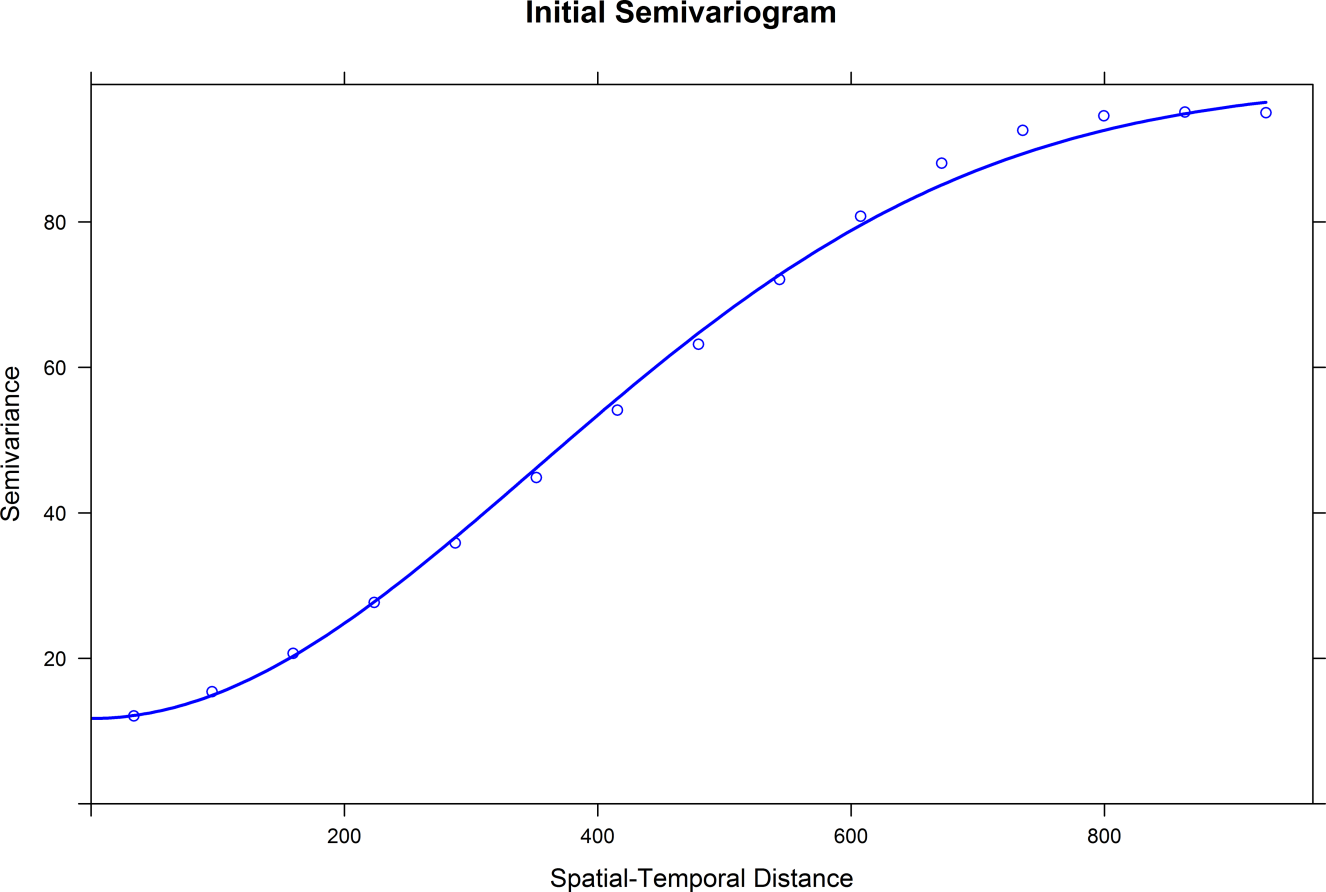
Imputation performance of proposed approach.

The proposed imputation method and the benchmark methods were tested on 11 different scenarios. The semivariogram is shown in Fig 3. Imputation results are shown in Table 3 and Fig 4. It can be seen from the table that the proposed imputation method is more accurate than the other two methods in most scenarios. Only when the missing rate is lower than 1%, the performance of the historical average method is better than the proposed imputation method. KNN method usually has the lowest imputation accuracy. This may be due to that there are only three features in this study for KNN to determine the nearest neighbors while KNN usually needs more than three features to obtain a reliable result [1].

**Table 3:** Performance of proposed approach.

Quantile	% Missing	MAD(Kr)	MAD(H)	MAD(Kk)	RMSE(Kr)	RMSE(H)	RMSE(Kk)
25%	1.0%	3.65	**3.06**	5.63	4.66	**4.16**	7.26
30%	1.4%	**2.63**	3.04	3.81	**3.46**	3.89	5.25
35%	1.8%	3.21	**3.03**	5.33	**4.22**	4.44	6.89
40%	2.8%	**2.78**	3.16	4.42	**3.86**	4.46	6.25
45%	4.4%	**3.00**	3.40	4.90	**4.07**	4.72	6.37
50%	6.0%	**2.85**	3.11	4.06	**3.88**	4.52	5.78
55%	8.3%	**2.84**	3.11	4.39	**3.96**	4.52	5.99
60%	13.9%	**2.85**	2.93	3.99	**3.93**	4.13	5.55
65%	20.7%	**2.70**	3.20	4.16	**3.71**	4.44	5.75
70%	31.1%	**2.77**	3.01	4.18	**3.87**	4.26	5.87
75%	36.1%	**2.66**	3.07	4.00	**3.71**	4.30	5.68

Note: Kr represents the proposed imputation method, H represents the historical average method, and Kk represents the KNN method.

## Conclusions

The paper presents a Kriging based spatiotemporal data imputation approach that is able to fully utilize the spatiotemporal information of the traffic data and that does not assume the data follow any distribution. As traffic flows from upstream to downstream, the traffic stream characteristics at a certain location are usually related to those at neighboring locations. So the traffic stream characteristics at upstream and downstream locations can be used to impute the missing value at a specific location. Besides, the traffic characteristics of a specific location at a certain time are also related to those of previous/future days or time periods. Therefore, a Kriging based imputation method that considers both temporal and spatial information is proposed. Compared with KNN and historical average, the proposed method has higher imputation accuracy in ten out of the eleven generated scenarios. Only when the data missing rate is lower than 1%, the performance of the historical average method is better than the proposed imputation method. It suggests that the historical average method is more suitable for the scenarios in which only a few data points are missing. This study also finds that the KNN method has the lowest imputation accuracy. The result of KNN may be more reliable when there are more features to determine the nearest neighbors are available.

## Supporting information

S1 Data Data used in this study. (RAR)
